# Self-Dehumanization Is Related to Worse Mental Health in Veterinarians

**DOI:** 10.3390/healthcare14010092

**Published:** 2025-12-31

**Authors:** Annalyse Ellis, Roxanne D. Hawkins, Sarah C. E. Stanton, Steve Loughnan

**Affiliations:** 1 Department of Psychology, School of Philosophy, Psychology, and Language Sciences, University of Edinburgh, Edinburgh EH8 9AD, UK; 2 Department of Clinical and Health Psychology, School of Health in Social Science, University of Edinburgh, Edinburgh EH8 9AG, UK

**Keywords:** veterinary, self-dehumanization, depression, anxiety, burnout, secondary traumatic stress

## Abstract

Background/Objectives: Veterinarians often experience poor mental health, including higher than typical rates of depression and anxiety. Self-dehumanization, which refers to the feeling of being less than human, may reflect an important yet neglected factor in poor veterinarian mental health. Methods: 201 veterinarians completed an online survey consisting of demographic questions, questions regarding their practice settings, and measures of self-dehumanization, depression, anxiety, burnout, and secondary traumatic stress. Results: About 10% of veterinarians reported self-dehumanization. Most veterinarians had clinically significant symptoms of depression and anxiety, as well as moderate levels of burnout and secondary traumatic stress. Burnout and secondary traumatic stress positively predicted anxiety, while burnout, secondary traumatic stress, and self-dehumanization positively predicted depression. Cross-sectional mediation analyses indicated that burnout and secondary traumatic stress both significantly mediated the links between self-dehumanization and anxiety, and self-dehumanization and depression. Conclusions: This study provides new insight into the role of self-dehumanization in the poor mental health of veterinarians, which has implications for the development of preventative measures.

## 1. Introduction

Veterinarians experience elevated rates of mental health concerns compared to the general population, including greater frequencies of depression, anxiety, psychological distress, and suicidality [1,2,3,4,5]. Potential contributing factors to poor mental health within the veterinary profession are well-researched [5], with a large body of work focusing on risk factors such as burnout and secondary traumatic stress. However, there are many other potential variables associated with veterinarian mental health that have not yet been explored. One such variable is self-dehumanization, which refers to the feeling of being less than human [6], and stems from an individual’s perception that they or the group to which they belong are lacking the essential qualities related to being human [7]. Self-dehumanization is experienced by other healthcare providers including nurses and doctors, and is linked to worse mental well-being outcomes such as negative emotions, low self-esteem, and increased anxiety [7,8,9,10]. However, self-dehumanization has not yet been studied in veterinarians to date despite similar probable risk factors (e.g., compassion fatigue) and coping mechanisms to deal with emotionally demanding roles, across healthcare professions [11]. The present work is the first to examine self-dehumanization amongst veterinarians. It aims to understand the associations between self-dehumanization, burnout, secondary traumatic stress, and the clinical mental health variables of anxiety and depression. Further, it aims to explore whether the relationships between self-dehumanization and clinical mental health variables are mediated by burnout and secondary traumatic stress in cross-sectional mediation analyses.

### 1.1. Veterinarian Mental Health

Veterinarian mental health has been an area of concern for researchers since the 1960s, with research expanding significantly in the 2000s [5]. It is widely recognized that veterinarians experience higher than average rates of mental illness, including general psychological distress [12,13], depression and other mood disorders [1,2,3,14,15], and anxiety disorders [2,12,16,17]. Existing studies indicate that the prevalence of depression in the overall population is around 5% [18], whereas several studies of depression in veterinarians indicate that prevalence ranges from between 10% and 30% [2,3,16,17,19]. The difference in anxiety prevalence between the overall population and veterinarians is even more acute: anxiety is experienced in around 4% of the general population [20], but rates range from 19.8% to more than 30% for veterinarians [2,16,17]. These statistics indicate a mental health crisis within the field of veterinary medicine. Further research examining the risk factors which perpetuate poor mental health amongst veterinarians is essential for the development of future preventive and protective measures.

There are several well-researched risk factors that may contribute to the poor mental health of veterinarians. For example, veterinarians often face long working hours and intense working schedules [21,22], which are associated with increased mortality risk, worse sleep, and worse mental health [23,24]. Some demographic factors, such as gender and age, are also associated with mental health; for example, women and younger veterinarians experience worse mental well-being [15]. Additionally, veterinarians also report financial stress [25], and often work with difficult or demanding clients [25,26,27], or bereaved clients [28], and these experiences are linked to client-related burnout and overall psychological distress [28,29]. Furthermore, veterinarians often encounter ethical dilemmas [30]; over half of veterinarians rank ethical dilemmas as the largest stressors within their jobs [31]. Finally, euthanasia is emotionally impactful for veterinarians, and its high frequency is associated with higher depression, higher compassion fatigue, and worse professional quality of life [32,33,34,35]. Thus, veterinarians experience many challenges in their working lives that are linked to higher stress levels and overall poor mental well-being. However, despite the large body of existing work on elements of the veterinary profession that are associated with worse veterinarian well-being, the role of self-dehumanization has not yet been explored, despite being highlighted as a risk factor in other healthcare professions.

### 1.2. Self-Dehumanization

Self-dehumanization is the feeling of being less than human, and an internalization of dehumanization [6]. Dehumanization encompasses animalistic dehumanization, which refers to seeing others as relatively more animal-like and lacking essential human characteristics; and mechanistic dehumanization, which refers to seeing others as cold, emotionless, and lacking autonomy [36]. The limited, but growing body of existing research indicates that self-dehumanization may be harmful to emotional health because of its impact on the perception of the self [6,7]. The experience of self-dehumanization is associated with numerous mental well-being concerns, across a variety of populations, but has been found to be particularly prevalent within healthcare professions [37]. Self-dehumanization has been highlighted as a key risk factor for poor mental health, and is associated with guilt, anger, sadness, anxiety, negative affect, suicidal thoughts, symptoms of distress, and a higher prevalence of psychological mood disorders [6,7,8,10,38].

There are several reasons why self-dehumanization may be experienced in veterinarians. Existing research on self-dehumanization within the human medical field indicates that healthcare professionals tend to self-dehumanize to a moderate extent as a coping mechanism to protect themselves from the emotional discomfort and psychological distress caused by witnessing suffering [11,39]. Physicians and veterinarians experience similar stressors and pressures [40], and therefore it is plausible that veterinarians may also self-dehumanize to cope with similar emotionally demanding work. Furthermore, self-dehumanization is linked to a sense of commodification within work, as well as self-pressure to maintain constant professionalism, and veterinarians face both of these concerns [41,42,43]. In sum, the existing literature suggests that veterinarians may also engage in self-dehumanization which could be contributing to the increased risk for mental health concerns within the profession.

### 1.3. Burnout and Secondary Traumatic Stress

Burnout is highly prevalent in veterinarians [12,14], with several existing studies indicating that between 15.6% and 51.6% of veterinarians experience burnout [44,45,46,47,48,49]. Burnout consists of emotional exhaustion, depersonalization, and a reduced sense of personal accomplishment [50]. There are several variables that may contribute to veterinarian burnout, including gender and age, with younger professionals and women reporting higher levels of burnout [44,51]. Burnout is associated with both depression and anxiety, and therefore likely also plays a large role in the overall mental well-being of veterinarians, potentially mediating the link between self-humanization and mental health symptoms [2,52].

Secondary traumatic stress refers to symptoms experienced by individuals in caring professions and first responders that imitate posttraumatic stress disorder symptomatology. These symptoms arise due to exposure to traumatic experiences of others [53]. Secondary traumatic stress is commonplace among veterinarians, with existing studies finding that the majority of veterinarians experience moderate or high levels [48,54,55], and with female veterinarians generally experiencing higher levels than male veterinarians [1,55]. Secondary traumatic stress is associated with several negative outcomes including personal distress [56], suicide risk [2], intention to leave the profession [57], and depression and anxiety [58]. Secondary traumatic stress has not yet been studied in relation to self-dehumanization, but could also be an important mediator in the link between self-humanization and poor mental health outcomes, in cross-sectional mediation analyses.

### 1.4. Study Aim

**Figure 1:**
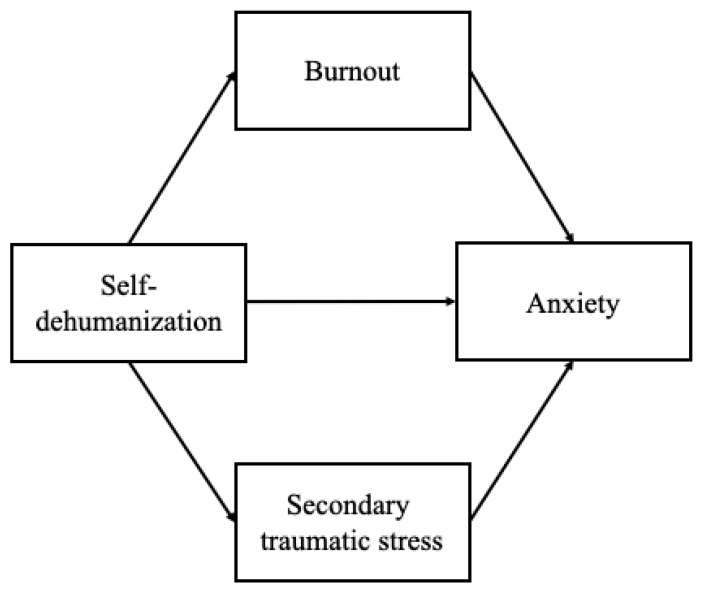
Visual representation of hypothesis with anxiety as the dependent variable.

**Figure 2:**
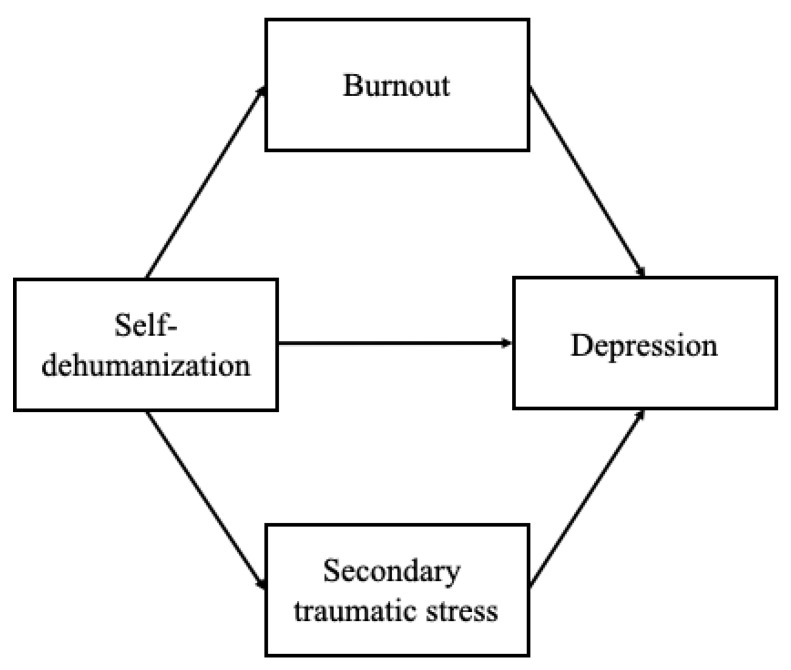
Visual representation of hypothesis with depression as the dependent variable.

This study aimed to investigate the associations between self-dehumanization, burnout, secondary traumatic stress, and clinically meaningful symptoms of anxiety and depression within the veterinary profession, using quantitative, cross-sectional methods. Our research questions were: (1) Do self-dehumanization, burnout, and secondary traumatic stress predict symptoms of anxiety and depression?; (2) Does burnout mediate the associations between self-dehumanization and symptoms of anxiety and depression?; and (3) Does secondary traumatic stress mediate the associations between self-dehumanization and symptoms of anxiety and depression? We hypothesized that self-dehumanization, burnout, and secondary traumatic stress would positively predict symptoms of anxiety and depression. We also hypothesized that burnout would mediate the associations between self-dehumanization and symptoms of anxiety and depression in cross-sectional mediation analyses, due to existing literature in which burnout mediated the relationships between self-dehumanization and depression in elite athletes [38]. Lastly, we hypothesized that secondary traumatic stress would also mediate the relationships between self-dehumanization and symptoms of anxiety and depression. Figure 1 and Figure 2 provide a visual representation of the hypotheses regarding the cross-sectional mediation analyses. We controlled for gender, age, years in the veterinary field, and overtime frequency in these analyses due to existing literature indicating that these variables are associated with well-being in veterinarians [15,21,22,59].

## 2. Materials and Methods

### 2.1. Participants

A total of 242 participants were recruited between October 2024 and December 2024 via a letter published by the British Veterinary Association (London, UK), flyers advertising the study posted to social media sites (Facebook [Menlo Park, CA, USA], X [Bastrop, TX, USA], and Reddit [San Francisco, CA, USA]), flyers advertising the study shared with the Royal (Dick) School of Veterinary Studies at the University of Edinburgh, and sharing flyers advertising the study with veterinary practices directly. The study flyer advertised that the study was focused on veterinarian mental well-being, and those interested were able to go to the link for the study or scan the QR code, both included on the flyer. Inclusion criteria for this study were being a currently practicing veterinarian. Forty-one participants were excluded for not completing the full survey, leaving a final analysis sample of 201 participants. See Table 1 for demographic information.

### 2.2. Materials

This study included measures of self-dehumanization, burnout, secondary traumatic stress, anxiety, and depression, a single item measuring frequency of overtime, demographic questions, and questions regarding veterinary practice setting. Mean scores were calculated for each measure. A measure of compassion satisfaction was also completed by participants, but this data was collected for a separate project. All materials are available on the Open Science Framework at https://osf.io/avnf7/ (accessed on 20 December 2025).

#### 2.2.1. Veterinarian Self-Dehumanization Scale

The 8-item Veterinarian Self-Dehumanization Scale was adapted from the Fontesse et al. [6] Self-Dehumanization Scale to be relevant to veterinarians by changing language in each item from “As an alcohol dependent person” to “As a veterinarian”. This scale was chosen due to acceptable internal consistency in previous work (α = 0.79) [6] and because the scale captures the salient aspects of self-dehumanization in a brief format. Participants indicated their agreement with each item (e.g., “As a veterinarian, I judge my worth as a human based on my successes or failures at work”) on a 7-point scale (1 = strongly disagree, 2 = disagree, 3 = somewhat disagree, 4 = neither agree nor disagree, 5 = somewhat agree, 6 = agree, 7 = strongly agree). Cronbach’s alpha was 0.80.

#### 2.2.2. Professional Quality of Life (ProQOL)

Twenty-nine items of the 30-item Professional Quality of Life Scale (ProQOL) [60] were utilized to measure burnout and secondary traumatic stress through separate subscales (one item of the original ProQOL in the secondary traumatic stress subscale, “I can’t recall important parts of my work with trauma victims” was excluded due to item phrasing that was a poor fit to the veterinary profession). This scale was chosen as the measure is widely used in veterinary mental health research, and due to its good construct validity and acceptable internal consistency in previous work (burnout α = 0.79, secondary traumatic stress α = 0.87) [1,2,5]. Participants indicated their agreement with each item (e.g., “Because of my work as a veterinarian, I have felt ‘on edge’ about various things”) on a 5-point scale (1 = never, 2 = rarely, 3 = sometimes, 4 = often, 5 = very often). Cronbach’s alpha was good for the burnout subscale (0.84) and the secondary traumatic stress subscale (0.83).

#### 2.2.3. Generalized Anxiety Disorder Assessment (GAD-7)

The 7-item GAD-7 [61] was utilized to measure anxiety. This scale was chosen due to its brevity, its measurement of anxiety symptoms, and its high internal consistency across a variety of populations (α = 0.92) [61]. Participants indicated their agreement with each item (e.g., “Worrying too much about different things”) on a 4-point scale (1 = not at all, 2 = several days, 3 = more than half the days, 4 = nearly every day). Cronbach’s alpha was 0.93.

#### 2.2.4. Patient Health Questionnaire (PHQ-9)

The 9-item PHQ-9 [62] was utilized to measure depression. This scale was chosen due to its wide use within veterinarian mental health studies [5], its brevity, its measurement of depression symptoms, and its high internal consistency across a variety of populations (α = 0.86) [63]. Participants indicated their agreement with each item (e.g., “Feeling tired or having little energy”) on a 4-point scale (1 = not at all, 2 = several days, 3 = more than half the days, 4 = nearly every day). Cronbach’s alpha was 0.91.

### 2.3. Procedure

This study received ethical approval (1-2425/2) from the University of Edinburgh School of Philosophy, Psychology, and Language Sciences Ethics Committee, for the collection of data from English-speaking participants both outside and within the United Kingdom, and was conducted in accordance with the Declaration of Helsinki. This study received no funding, and all materials are available on the Open Science Framework at https://osf.io/avnf7/ (accessed on 20 December 2025). The survey was hosted on Qualtrics. Participants were provided with information regarding the study, and upon providing consent, they were asked to complete the survey. Scales were randomized within the survey, as were the items in each scale. At the end of the survey, participants were debriefed and provided with location-relevant information on mental health resources, based on the country in which they reported living. These resources included contact information for crisis support services, due to the nature of questions asked in the survey, including one question related to suicidality included in the PHQ-9.

### 2.4. Data Analysis and Analytic Plan

Prior to participant recruitment, an a priori power analysis was conducted using G*Power Version 3.1.9.6 (Düsseldorf, Germany) [64]. Results indicated that 160 participants were required to achieve 80% power for detecting a medium effect. All data analysis was conducted using RStudio version 2024.09 and version 2025.05.0 (Posit, Boston, MA, USA) [65]. The following R packages were utilized: psych version 2.3.6 (Evanston, IL, USA) [66], lavaan version 0.6-16 (Gent, Belgium) [67], dplyr version 1.1.2 (Boston, MA, USA) [68], car version 3.1-2 (Thousand Oaks, CA, USA) [69], and lmtest version 0.9-40 (Zurich, Switzerland) [70]. Planned analyses included correlations among all variables; descriptive statistics of all variables; analysis of the symptom severity of depression, anxiety, burnout, and secondary stress; hierarchical regression models with anxiety and depression as dependent variables; and cross-sectional mediation models with self-dehumanization as the predictor variable, secondary traumatic stress and burnout as mediators, and depression and anxiety as dependent variables.

## 3. Results

### 3.1. Correlations, Descriptives, and Symptom Severity

Table 2 contains correlations and descriptive statistics. Anxiety, depression, secondary traumatic stress, burnout, and self-dehumanization displayed significant patterns of correlation with all other variables.

Table 3 contains information regarding symptom severity and cut off values regarding anxiety, depression, secondary traumatic stress, and burnout. Nearly two-thirds of the participants reported clinically significant levels of anxiety and depression. Additionally, more than two-thirds of participants reported moderate or severe burnout and secondary traumatic stress. It is important to note that while the instruments used are strong indicators of the presence of these mental health concerns, psychological disorders are never diagnosed with just one questionnaire, and therefore the results reported here are should not be understood as clinical diagnoses.

The mean score for self-dehumanization (2.63) was compared to the scale midpoint (4.00), using a single sample *t*-test. The scale midpoint was used for this analysis as scores above the midpoint indicate high levels of self-dehumanization, while scores below the midpoint indicate low levels of self-dehumanization. The single sample *t*-test revealed that the mean score was significantly lower than the scale midpoint (*t*(200) = −19.37, *p* < 0.001), indicating that a majority of veterinarians included in this sample do not engage in high levels of self-dehumanization. However, 10.4% of participants (21 participants) did endorse higher levels of self-dehumanization than the midpoint level, indicating high self-dehumanization for these participants.

### 3.2. Regressions

Two three-step hierarchical multiple regressions were conducted to examine whether self-dehumanization, burnout, and secondary traumatic stress are predictors of anxiety and depression, while controlling for gender, years in the veterinary field, and overtime frequency, due to their roles in veterinarian well-being [1,44,51,54,55]. Age was excluded as a control variable because of collinearity concerns with years in the veterinary field, which was retained as it was considered the more relevant variable. Prior to analysis, all predictor variables except for gender were grand mean-centered; and gender was coded for analyses (0 = men, 1 = women).

Table 4 reports the results of the models in which anxiety is the dependent variable. Prior to these analyses, assumptions of multiple regression were evaluated. The assumption of linearity was met. Normality of residuals was assessed and indicated a deviation from normality, suggesting a positive skew in the dependent variable. To address this issue, the dependent variable was log-transformed, which improved the distribution of residuals. Tests for multicollinearity indicated no serious concerns and results of the Breusch-Pagan Test suggested homoscedasticity. All analyses were then conducted using the transformed outcome variable.

In Step 1, demographic variables (gender, years in field, and frequency of overtime) accounted for 21.0% of variance in anxiety. The results showed that the first model was significant *F*(3, 197) = 17.48, *p* < 0.001, with both gender and frequency of overtime significantly and positively predicting anxiety, and years in field significantly and negatively predicting anxiety. In Step 2, the addition of self-dehumanization explained an additional 13.9% of variance, *F_change_*(1, 196) = 70.12, *p* < 0.001. In this model, gender and frequency of overtime continued to significantly and positively predict anxiety, as did self-dehumanization, while years in field continued to significantly and negatively predict anxiety. In Step 3, the inclusion of burnout and secondary traumatic stress accounted for an additional 26.6% of variance, *F_change_*(2, 194) = 66.87, *p* < 0.001. In this model, only burnout and secondary traumatic stress were significant positive predictors of anxiety.

Table 5 reports the results of the models in which depression is the dependent variable. Prior to these analyses, assumptions of multiple regression were evaluated. The assumption of linearity was not met. Normality of residuals was assessed, which indicated a deviation from normality, suggesting a positive skew in the dependent variable. To address this issue, the dependent variable was again log-transformed, which improved the distribution of residuals. The assumption of linearity was retested and met. Tests for multicollinearity indicated no serious concerns, and results of the Breusch-Pagan Test suggested homoscedasticity. All analyses were then conducted using the transformed outcome variable.

In Step 1, demographic variables (gender, years in field, and frequency of overtime) accounted for 22.4% of variance in depression. The results showed that the first model was significant *F*(3, 197) = 19.00, *p* < 0.001, with frequency of overtime significantly and positively predicting depression, and years in field significantly and negatively predicting depression. In Step 2, the addition of self-dehumanization explained an additional 18.9% of variance, *F_change_*(1, 196) = 104.17, *p* < 0.001. In this model, frequency of overtime continued to significantly and positively predict depression, as did self-dehumanization, while years in field significantly and negatively predicted depression. In Step 3, the inclusion of burnout and secondary traumatic stress accounted for an additional 23.6% of variance, *F_change_*(2, 194) = 65.45, *p* < 0.001. In this model, self-dehumanization, burnout and secondary traumatic stress were significant positive predictors of depression, while years in field was a significant negative predictor of depression.

### 3.3. Cross-Sectional Mediation

**Figure 3:**
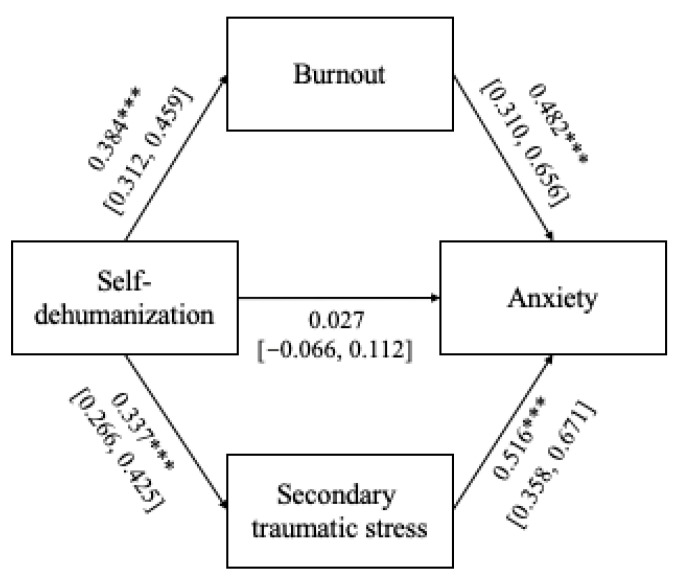
Parallel path mediation models with anxiety as the dependent variable. Note: *** *p* < 0.001.

**Figure 4:**
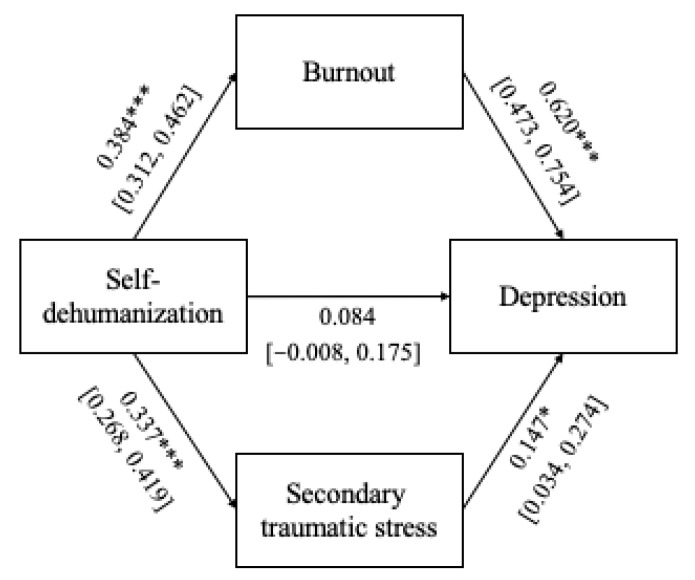
Parallel path mediation models with depression as the dependent variable. Note: * *p* < 0.05, *** *p* < 0.001.

Two parallel path mediation models were used to test the significance of both direct and indirect effects via burnout and secondary traumatic stress of self-dehumanization on both anxiety and depression using bootstrapping with 1000 samples and 95% confidence intervals. The total indirect effects of self-dehumanization on anxiety (β = 0.359, 95% CI = 0.284–0.447) were found to be statistically significant. The total indirect effects of self-dehumanization on depression (β = 0.288, 95% CI = 0.220–0.358) were also found to be statistically significant. Figure 3 and Figure 4 report the results of the cross-sectional path mediation models.

## 4. Discussion

This study is the first to systematically investigate the role of self-dehumanization in veterinarians’ mental well-being. We found that self-dehumanization is an important but previously unknown element of veterinarians’ mental health. The results for the prevalence of burnout, secondary traumatic stress, anxiety, and depression within the veterinary profession align with previous work [1,2,12,14,17,55].

### 4.1. The Role of Self-Dehumanization

Results regarding the role of self-dehumanization align with existing work regarding self-dehumanization within other populations, while providing novel insight into the field of veterinarian well-being. Existing literature in other fields indicates that higher levels of self-dehumanization are associated with worse mental well-being [8,10,38]. This study found that self-dehumanization correlates positively with overtime frequency, burnout, secondary traumatic stress, depression, and anxiety. The present work also found that self-dehumanization is a significant positive predictor of anxiety, but that when secondary traumatic stress and burnout were added to this model, self-dehumanization was no longer a significant predictor. Conversely, self-dehumanization significantly predicted depression regardless of the presence of secondary traumatic stress and burnout within the model. This finding indicates that self-dehumanization plays a principal and unique role in the well-being of veterinarians, especially related to depression. Although only 10.4% of participants reported experiencing high self-dehumanization, this is still a substantial subset of the veterinary population, and therefore the role of self-dehumanization should not be overlooked in future research. It is possible that the subset of veterinarians experiencing high self-dehumanization may feel more burdened by client demands, commodification of their work, and pressures to maintain professionalism [41,42,43], or by the suffering of their patients [11,39]. Additionally, many veterinarians report that their professions are central to their identities and self-concept [71]. It is plausible that veterinarians experiencing higher levels of self-dehumanization also view their occupations as more central to their identity, to the detriment of other aspects of their identity as humans.

### 4.2. Veterinarian Mental Well-Being

High prevalence of poor mental well-being was found in this population, to an even greater severity than indicated in existing literature. Of the veterinarians included in this sample, 62.7% had clinically significant depression symptoms, and 61.2% had clinically significant anxiety symptoms, encompassing mild, moderate, moderately severe (depression only), and severe presentations of these mental health concerns. The existing literature indicates that prevalence of depression for veterinarians tends to range from more than 10% to nearly 30% [2,3,16,17,19], while the prevalence of anxiety tends to range from 19.8% to more than 30% [2,16,17]. These differences may be due to the use of different cut off scores within the studies [72], or differences in reporting (e.g., reporting all clinical levels of depression or anxiety versus only severe levels of the mental health concerns). The present study reports all clinical levels of depression and anxiety because even mild presentations of these mental health concerns can have important overall well-being impacts [73], but some of the other previous studies only report severe clinical levels, causing prevalence estimates of depression and anxiety to appear lower.

Burnout and secondary traumatic stress were also prevalent within our veterinarian population, with 66.2% of participants reporting moderate levels of burnout, and 68.7% reporting moderate levels of secondary traumatic stress. Interestingly, few participants reported high levels of burnout or secondary traumatic stress, while existing research has found a much higher prevalence of high scores in both of these variables [45,48,54]. Burnout and secondary traumatic stress both displayed positive correlations with overtime frequency, depression, anxiety, and one another. Additionally, burnout was a significant predictor of both depression and anxiety, which aligns with existing literature [2,52], while secondary traumatic stress was a significant predictor of anxiety only. Surprisingly, secondary traumatic stress did not significantly predict depression, although previous work has found that secondary traumatic stress may predict risk of suicide [2], and is associated with personal distress [56]. It is uncertain why secondary traumatic stress did not predict depression in this study, and more research is needed to explore the association between these two variables in veterinarians.

### 4.3. Mediating Role of Burnout and Secondary Traumatic Stress on the Relationship Between Self-Dehumanization and Clinical Mental Well-Being

The results of cross-sectional mediation analyses found that burnout and secondary traumatic stress mediated the links between self-dehumanization and depression, and self-dehumanization and anxiety. These findings indicate that self-dehumanization is linked to both burnout and secondary traumatic stress, which then may manifest as increased clinical mental health concerns. These results are novel within this population, and merit further exploration in this field. However, past research does link burnout and secondary traumatic stress to depression and anxiety in both veterinarians and other populations [2,52]; and self-dehumanization to depression and anxiety in other populations [7,8,10]. A key implication of these findings relates to the potential importance of reducing the experience of self-dehumanization in veterinarians in order to influence both burnout and secondary traumatic stress, as well as the prevalence of clinical mental health concerns.

### 4.4. Limitations and Future Directions

There are a few key limitations related to this study, as well as future directions for the field. First, this study is limited by potential sample self-selection bias. Because participants did not receive compensation, the veterinarians who participated may have been particularly interested in this subject area, and results therefore may not be generalizable across all veterinarians. This potential sample self-selection bias may be responsible for the greater prevalence of clinically significant levels of anxiety and depression found in this study than in past studies, and therefore this finding should be treated with caution. Additionally, sampling was non-random; participants were recruited via a letter published by the British Veterinary Association, social media sites, the Royal (Dick) School of Veterinary Studies at the University of Edinburgh, and contacting veterinary practices directly. Three further limitations of this study are related to the sample: the veterinarians included in this study largely worked in small animal (92.0%), non-emergency practice settings, mostly (75.6%) worked a first shift schedule, and were primarily (70.1%) from the United Kingdom. Unfortunately, this limited our ability to make meaningful comparisons across practice types and shift schedules, and limited generalizability of results. Future directions of this work could include a comparison of veterinarians in different practice settings, as there may be differences in mental well-being. For example, veterinarians working with equines, a mixture of small and large animals, and production animals face elevated risks [74], which play a role in mental well-being. Additionally, veterinarians who spend most of their time working with cats and dogs have higher levels of burnout and secondary traumatic stress [54]. Furthermore, there is a shortage of veterinarians working in emergency medicine, in part due to the perceived stress of working in such a setting, but also due to challenges regarding scheduling, as emergency medicine typically requires working shifts outside of a traditional first shift schedule [21]. Future work could further explore the roles that practice settings and shift schedules play in veterinarian mental well-being, addressing the limitations of the present work.

Another key limitation of the present work is its cross-sectional design. Neither directionality nor causality cannot be assumed regarding the associations among the variables included in this study, and no causal statements can be made related to the findings of this work. There is a shortage of longitudinal work in this field, and future work could explore these variables over time, addressing the limitations of this study related to causality and directionality. This would be a particularly interesting area of future study because past literature indicates that veterinarians who have been in the field longer typically have better mental well-being, but existing work has been cross-sectional, not taking into account that perhaps veterinarians with worse mental well-being are exiting the field [15]. Another area for future research is the association between identity centrality (i.e., viewing their profession as a key element of their self-concept) [71], identity conflict, and self-dehumanization. Research regarding identity centrality within veterinarians has found that veterinarians experience both professional identity centrality [71,75] as well as identity conflicts between their ideal (e.g., most professional) and real selves [76]. It is possible that veterinarians who experience both high levels of professional identity centrality and frequent identity conflicts could also experience self-dehumanization related to aspects of their identities outside of their professional identities. Future research could explore the associations between professional identity centrality, identity conflict, and self-dehumanization within veterinarians. Existing work indicates that personality traits play a role in veterinarian well-being [77], and future work could explore the relationships between personality traits as they relate to veterinarians’ experience of self-dehumanization.

Finally, future work could explore the development of interventions aimed at reducing self-dehumanization for veterinarians. The need for mental health support for veterinarians is receiving increased attention, leading to the development of new strategies to combat mental health risks, such as universities incorporating mental well-being modules into their veterinary students’ education and the growth and development of the veterinary social work field [78,79]. Additionally, research on dehumanization highlights strategies to combat self-dehumanization [80], including self-forgiveness [81], and increasing perceived humanization by others [82]. Future work could explore whether mental health interventions such as Cognitive Behavioral Therapy or Compassion-Focused Therapy could help reduce self-dehumanization, via developing self-compassion and self-forgiveness and reframing distorted thought patterns [83,84]. These interventions could potentially be integrated into existing programs focused on addressing veterinary mental health in order to address this facet of poor mental well-being.

## 5. Conclusions

The aim of the present work was to explore the role of self-dehumanization in veterinarians’ mental well-being. Importantly, the findings of this study align with existing work, which has found that veterinarians experience poor mental well-being, and also provide new insight to the field regarding the role of self-dehumanization. These results indicate that self-dehumanization plays an important role in the mental well-being of veterinarians, suggesting a key focus for future strategies to reduce self-dehumanization in the veterinary profession. Future longitudinal work is needed in order to better understand the relationships among self-dehumanization and the mental well-being variables included in this study. The findings of the present work have implications for further research related to interventions aimed at reducing self-dehumanization and improving the well-being of veterinarians at a systemic level.

## Figures and Tables

**Table 1:** Demographics table. SD (standard deviation).

	Overall (*N* = 201)
Gender	
Woman	147 (73.1%)
Man	54 (26.9%)
Transgender	
No	197 (98.0%)
Yes	3 (1.5%)
Don’t know	1 (0.5%)
Sexual Orientation	
Heterosexual, Straight	163 (81.1%)
Bisexual, Pansexual	17 (8.5%)
Gay	10 (5.0%)
Queer	4 (2.0%)
Lesbian	3 (1.5%)
Demisexual	3 (1.5%)
Asexual	1 (0.5%)
Ethnicity	
White, Caucasian, Anglo	178 (88.6%)
Not represented	6 (3.0%)
Hispanic, Latino/a, Chicano/a	4 (2.0%)
Mixed or Multiple Ethnic Groups	4 (2.0%)
Southeast Asian	4 (2.0%)
South Asian	3 (1.5%)
East Asian	2 (1.0%)
Country	
United Kingdom	141 (70.1%)
United States	23 (11.4%)
Australia	19 (9.5%)
Canada	9 (4.5%)
New Zealand	7 (3.5%)
Argentina	1 (0.5%)
Germany	1 (0.5%)
Practice Setting	
Small animal practice	185 (92.0%)
Exotic animal practice	11 (5.5%)
Emergency practice	10 (5.0%)
Equine-only practice	8 (4.0%)
Large animal practice	3 (1.5%)
Laboratory	2 (1.0%)
Animal shelter	2 (1.0%)
Wildlife	2 (1.0%)
Food supply	1 (0.5%)
Shift Schedule	
First shift	152 (75.6%)
Rotating shift schedule	41 (20.4%)
Second shift	4 (2.0%)
Third shift	4 (2.0%)
Age	
Mean (SD)	40.9 (11.8)
Median [Min, Max]	37.0 [23.0, 82.0]

**Table 2:** Correlations and descriptive statistics.

	Mean	SD	Age	Years in Field	Overtime Frequency	Self- Dehumanization	Burnout	Secondary Traumatic Stress	Depression	Anxiety
**Age**	40.88	11.80		0.972 ***	−0.089	−0.163 *	−0.266 ***	−0.273 ***	−0.320 ***	−0.300 ***
**Years in field**	16.47	11.88			−0.095	−0.163 *	−0.275 ***	−0.274 ***	−0.331 ***	−0.307 ***
**Overtime frequency**	3.37	1.24				0.206 **	0.352 ***	0.295 ***	0.303 ***	0.281 ***
**Self-dehumanization**	21.01	8.04					0.571 ***	0.482 ***	0.523 ***	0.457 ***
**Burnout**	26.05	6.76						0.649 ***	0.748 ***	0.679 ***
**Secondary traumatic stress**	21.72	6.33							0.582 ***	0.691 ***
**Depression**	16.77	6.43								0.784 ***
**Anxiety**	14.69	5.94								

Note: * *p* < 0.05, ** *p* < 0.01, *** *p* < 0.001.

**Table 3:** Symptom severity.

Depression	Cut Off Values	*N* (%)
No clinical significance	9–13	75 (37.3%)
Mild	14–18	59 (29.4%)
Moderate	19–23	37 (18.4%)
Moderately severe	24–28	17 (8.5%)
Severe	29 and above	13 (6.5%)
**Anxiety**		
No clinical significance	7–11	78 (38.8%)
Mild	12–16	54 (26.9%)
Moderate	17–21	30 (14.9%)
Severe	22 and above	39 (19.4%)
**Burnout**		
Low	10–22	67 (33.3%)
Moderate	23–41	133 (66.2%)
High	42 and above	1 (0.5%)
**Secondary Traumatic Stress**		
Low	9–17	61 (30.3%)
Moderate	18–36	138 (68.7%)
High	37 and above	2 (1.0%)

**Table 4:** Three-step hierarchical multiple regression with anxiety as the dependent variable.

	Step 1 β [95% CI]	Step 2 β [95% CI]	Step 3 β [95% CI]
(Intercept)	0.534 *** [0.436–0.631]	0.554 *** [0.465–0.643]	0.605 *** [0.535–0.675]
Gender	0.174 ** [0.060–0.288]	0.147 ** [0.042–0.251]	0.076 [−0.006–0.159]
Years in field	−0.009 *** [−0.013–−0.005]	−0.007 *** [−0.011–−0.003]	−0.003 [−0.006–0.000]
Frequency of overtime	0.084 *** [0.044–0.125]	0.060 ** [0.023–0.098]	0.008 [−0.023–0.039]
Self-dehumanization		0.155 *** [0.108–0.202]	0.019 [−0.025–0.062]
Burnout			0.227 *** [0.150–0.304]
Secondary traumatic stress			0.218 *** [0.148–0.287]
R2/R2 adjusted	0.210/0.198	0.349/0.336	0.615/0.603

Note: ** *p* < 0.01, *** *p* < 0.001.

**Table 5:** Three-step hierarchical multiple regression with depression as the dependent variable.

	Step 1 β [95% CI]	Step 2 β [95% CI]	Step 3 β [95% CI]
(Intercept)	0.485 *** [0.397–0.572]	0.506 *** [0.429–0.583]	0.530 *** [0.470–0.591]
Gender	0.095 [−0.008–0.198]	0.066 [−0.024–0.156]	0.033 [−0.039–0.104]
Years in field	−0.010 *** [−0.014–−0.006]	−0.008 *** [−0.011–−0.005]	−0.004 ** [−0.007–−0.002]
Frequency of overtime	0.083 *** [0.046–0.120]	0.058 ** [0.025–0.091]	0.012 [−0.015–0.039]
Self-dehumanization		0.164 *** [0.123–0.205]	0.045 * [0.007–0.083]
Burnout			0.306 *** [0.239–0.373]
Secondary traumatic stress			0.063 * [0.002–0.123]
R2/R2 adjusted	0.224/0.213	0.413/0.401	0.649/0.638

Note: * *p* < 0.05, ** *p* < 0.01, *** *p* < 0.001.

## Data Availability

The data presented in this study are available on request from the corresponding author The data are not publicly available due to privacy reasons.
